# Improved long-term expression from helper virus-free HSV-1 vectors packaged using combinations of mutated HSV-1 proteins that include the U_L_13 protein kinase and specific components of the VP16 transcriptional complex

**DOI:** 10.1186/1471-2199-10-58

**Published:** 2009-06-16

**Authors:** Meng Liu, Xiaodan Wang, Alfred I Geller

**Affiliations:** 1 Department of Neurology, West Roxbury VA Hospital/Harvard Medical School, W. Roxbury, MA 02132, USA

## Abstract

**Background:**

Herpes Simplex Virus (HSV-1) gene expression is thought to shut off recombinant gene expression from HSV-1 vectors; however, in a helper virus-free HSV-1 vector system, a number of promoters support only short-term expression. These results raise the paradox that recombinant gene expression remains short-term even in the absence of almost all (~99%) of the HSV-1 genome, HSV-1 genes, and HSV-1 gene expression. To resolve this paradox, we hypothesized that specific proteins in the HSV-1 virus particle shut off recombinant gene expression. In two earlier studies, we examined the effects on recombinant gene expression of packaging vectors using specific mutated HSV-1 proteins. We found that vectors packaged using mutated U_L_13 (a protein kinase), or VP16, or U_L_46 and/or U_L_47 (components of the VP16 transcriptional complex) supported improved long-term expression, and vectors packaged using mutated U_L_46 and/or U_L_47 also supported improved gene transfer (numbers of cells at 4 days). These results suggested the hypothesis that specific proteins in the HSV-1 particle act by multiple pathways to reduce recombinant gene expression. To test this hypothesis, we examined combinations of mutated proteins that included both U_L_13 and specific components of the VP16 transcriptional complex.

**Results:**

A HSV-1 vector containing a neuronal-specific promoter was packaged using specific combinations of mutated proteins, and the resulting vector stocks were tested in the rat striatum. For supporting long-term expression, the preferred combination of mutated HSV-1 proteins was mutated U_L_13, U_L_46, and U_L_47. Vectors packaged using this combination of mutated proteins supported a higher efficiency of gene transfer and high levels expression for 3 months, the longest time examined.

**Conclusion:**

Vector particles containing this combination of mutated HSV-1 proteins improve recombinant gene expression. Implications of these results for strategies to further improve long-term expression are discussed. Moreover, long-term expression will benefit specific gene therapy applications.

## Background

Herpes Simplex Virus (HSV-1) vectors are an attractive system for gene transfer into neurons, but the limited levels of long-term expression remain a significant problem. The numbers of cells expressing recombinant gene products display large decreases within the first few weeks after gene transfer, using either plasmid (amplicon) or recombinant HSV-1 vectors that contain specific viral or neuronal-specific cellular promoters (reviewed in [1]). Interestingly, using HSV-1 plasmid vectors, two neuronal type-specific promoters (tyrosine hydroxylase (TH) or preproenkephalin), and chimeric promoters that contain 5' upstream sequences from either of these promoters, support significant levels of long-term expression (2–14 months) [1-8]. Although the levels of expression display a significant initial decrease, long-term expression is relatively stable. Consequently, identifying the mechanisms responsible for the initial decrease in expression may provide a foundation for the rational design of HSV-1 vectors that support long-term expression.

The reduction in recombinant gene expression might be caused by the mechanisms that repress HSV-1 gene expression in HSV-1 latency, focusing attention on HSV-1 gene regulation. Supporting a role for HSV-1 gene regulation in reducing recombinant gene expression, helper virus-containing HSV-1 vector systems cause significant cytopathic effects and an inflammatory response, and most of these side effects are caused by HSV-1 gene expression from the helper virus [9-11]. Consistent with these results, helper virus-free HSV-1 vectors cause substantially less side effects than those observed using helper virus-containing systems [12,13]. However, helper virus-free HSV-1 vectors support minimal, if any, improvement in long-term expression, using a number of viral or neuronal-specific cellular promoters [7,12]. Thus, paradoxically, expression from these promoters remains short-term, even in the absence of almost all (~99%) of the HSV-1 genome, the latency associated transcript (LAT) gene, HSV-1 genes, and HSV-1 gene expression.

To explain this paradox, we proposed that specific proteins in the HSV-1 particle reduce recombinant gene expression [14]. Specific proteins in the HSV-1 particle have prominent roles in the process of the virus taking control of the cell's biosynthetic machinery (reviewed in [15]). Thus, specific proteins in the HSV-1 particle might alter recombinant gene expression. In an initial study, we examined the effects on recombinant gene expression of five proteins that affect the HSV-1 particle [14]. We found that packaging vectors using a mutated *vhs *or U_S_11 resulted in minimal changes in recombinant gene expression; packaging vectors using a mutated U_S_3 resulted in improved gene transfer (number of cells at 4 days); and packaging vectors using a mutated U_L_13, a protein kinase, or VP16 supported long-term expression (2 months).

These results suggested the hypothesis that specific proteins in the HSV-1 particle act by multiple pathways to reduce recombinant gene expression. Thus, interfering with multiple pathways might support higher levels of recombinant gene expression than those obtained by interfering with a single pathway. Specifically, packaging vectors using both mutated U_L_13 and mutated VP16 might support higher levels of long-term expression than those obtained by packaging vectors using either mutated U_L_13 or mutated VP16.

Unfortunately, packaging using mutated VP16 lowered the titers ~100-fold, and packaging using both mutated VP16 and other mutated HSV-1 proteins further lowered the titers to levels that cannot support *in vivo *gene transfer experiments. To improve the titers, and to explore the mechanism by which VP16 reduces long-term expression, we examined the effects on recombinant gene expression of two HSV-1 proteins, U_L_46 and U_L_47, that modulate the activity of VP16 [16-18]. Vectors packaged using an HSV-1 cosmid set harboring a deletions of U_L_46, or U_L_47, or both, supported both improved gene transfer and long-term expression [19]. Vectors packaged using an HSV-1 cosmid set harboring deletions in both U_L_46 and U_L_47 supported larger improvements in recombinant gene expression compared to vectors packaged using an HSV-1 cosmid set harboring a deletion of either gene alone. Also, packaging using an HSV-1 cosmid set harboring the deletion of both U_L_46 and U_L_47 supported higher titers than packaging using a mutated VP16. Of note, a second approach to improve the titers using mutated VP16 has been reported: A recombinant HSV-1 vector containing a mutated VP16 was grown to high titer in the presence of equine herpesvirus (EHV-1) gene 12 [20], the EHV-1 homolog of HSV-1 VP16. EHV-1 gene 12 transactivates HSV-1 promoters, thereby restoring the titers to levels similar to those obtained using wild type (wt) VP16. However, EHV-1 gene 12 is not incorporated into HSV-1 particles, so EHV-1 gene 12 is unlikely to reduce recombinant gene expression.

In the present study, we tested the hypothesis that specific proteins in the HSV-1 particle act by multiple pathways to reduce recombinant gene expression. We examined the long-term expression supported by a HSV-1 vector that contained a neuronal-specific promoter and was packaged using an HSV-1 cosmid set harboring combinations of mutated proteins that included U_L_13 and specific proteins in the VP16 transcriptional complex. The preferred combination was mutated U_L_13, U_L_46, and U_L_47, and this packaging condition improved both gene transfer and long-term expression.

## Results

### HSV-1 vectors packaged using specific combinations of mutated HSV-1 proteins

In this study, we tested the hypothesis that specific proteins in the HSV-1 particle act by multiple pathways to reduce recombinant gene expression. We packaged pNFHlac [7], which contains the neurofilament heavy gene (NF-H) promoter, using two combinations of mutated HSV-1 proteins that included a point mutation in U_L_13 (U_L_13g, abolishes protein kinase activity [14]) and a mutated protein in the VP16 transcriptional complex, either a linker insertion mutation in VP16 (VP16*in*14 [21,22]) or a deletion of both U_L_46 and U_L_47 (ΔU_L_46/47 [16,17,19]). Packagings that used VP16*in*14 also contained EHV-1 gene 12 to improve the titers [20]. Control packaging conditions included i) U_L_13g, ii) VP16*in*14 and EHV-1 gene 12, iii) ΔU_L_46/47, or iv) standard conditions (wt, no mutated HSV-1 proteins), as the negative control. The numbers of infectious vector particles (IVP/ml) in these vector stocks were determined by 5-bromo-4-chloro-3-indoyl-β-D-galactopyranoside (X-gal) staining at 24 hours after transduction of baby hamster kidney (BHK) fibroblast cells. The results (Table 1) showed that pNFHlac/vp16in14, gene12, ul13g (nomenclature, vector followed by the mutated proteins used in packaging) reduced the titers 7.2-fold, and the other packaging conditions supported titers that were similar (≤ 3-fold reduction) to those obtained using standard packaging conditions (pNFHlac/wt). Next, we determined the titers of vector genomes (VG/ml); we isolated DNA from the vector stocks and performed PCR using primers from the Lac Z gene [14]. As a measure of the packaging efficiency, we calculated the ratio of the physical titer to the biological titer (VG/IVP) for each vector stock. The results (Table 1) showed that packaging pNFHlac in the presence of any of these mutated HSV-1 proteins, alone or in combination, resulted in similar ratios of the physical titer to the biological titer compared to packaging using standard conditions. Thus, these mutated HSV-1 proteins, alone or in specific combinations, did not cause large changes in the packaging efficiency.

**Table 1:** The numbers of X-gal positive striatal cells from rats sacrificed at various times after microinjection of pNFHlac packaged using specific combinations of mutated HSV-1 proteins

	Purified titers^a^			Average X-gal positive cells per rat Time after gene transfer					Relative efficiency of gene transfer^b^
	
Packaging condition	VG/ml	IVP/ml	VG/IVP	4 days	2 wks	1 month	2 months	3 months	
^c^Δul46&47, ul13g	6.4 × 10^7^	5.0 × 10^6^	10.1	1023 ± 23	680 ± 49	480 ± 11	319 ± 5	155 ± 30	5.7
VP16in14, gene12, ul13g	7.6 × 10^6^	7.2 × 10^5^	10.1	201 ± 8	119 ± 9	65 ± 6	44 ± 4	23 ± 6	2.3
VP16in14, gene12	1.2 × 10^7^	1.8 × 10^6^	6.7	343 ± 17	104 ± 4	65 ± 15	36 ± 8	16 ± 2	1.6
ul13g	2.0 × 10^7^	1.8 × 10^6^	11.1	393 ± 17	197 ± 6	99 ± 16	47 ± 19	19 ± 5	1.8
Δul46&47^d^	3.1 × 10^7^	3.0 × 10^6^	10	2,532 ± 93	980 ± 88	616 ± 58	315 ± 17	92 ± 11	9.9
wt^d^	6.3 × 10^7^	5.2 × 10^6^	12	445 ± 39	63 ± 9	12 ± 2	0 ± 0	0 ± 0	1.0

Three rats were analyzed for condition and time point; the means ± SDs are shown.

^a^The titers of each vector stock after purification and concentration. VG/ml is vector genomes/ml; IVP/ml is infectious vector particles/ml.

^b^The efficiency of gene transfer is the number of positive cells at 4 days/the amount of vector injected. The relative efficiency of gene transfer is the efficiency of gene transfer with a specific condition/the efficiency of gene transfer with wt packaging.

^c^Vector stock diluted 3.3-fold before gene transfer.

^d^For these two packaging conditions, the titers and the X-gal cell counts for 4 days through 2 months were previously reported in [19] and are included here only to support comparisons. The X-gal cell counts at 3 months have not been previously reported.

### The levels of long-term expression supported by HSV-1 vectors packaged using specific combinations of mutated HSV-1 proteins

**Figure 1:**
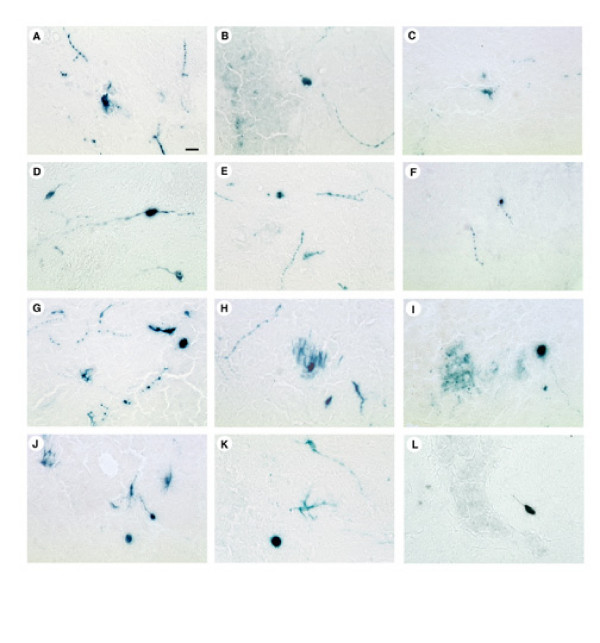
**X-gal positive striatal cells from rats that received microinjections of pNFHlac packaged using specific combinations of mutated HSV-1 proteins**. The rats were sacrificed at 4 days, 2 months, or 3 months after gene transfer. **A-C**. pNFHlac/ul13g; 4 days after gene transfer (A), 2 months (B), and 3 months (C). **D-F**. pNFHlac/VP16in14, gene12; 4 days (D), 2 months (E), and 3 months (F). **G-I**. pNFHlac/Δul46&47, ul13g; 4 days (G), 2 months (H), and 3 months (I). **J-L**. pNFHlac/VP16in14, gene12, ul13g; 4 days (J), 2 months (K), and 3 months (L). These high power views show X-gal positive cell bodies and proximal processes. Scale bar: 25 μm.

These vector stocks were microinjected into the striatum, the rats were sacrificed 4 days, or 2 weeks, or 1, 2, or 3 months after gene transfer, and X-gal staining was performed. Control rats that received PBS lacked X-gal positive striatal cells, but faint X-gal staining was observed in small numbers of cells that lined the brain vasculature (not shown). Sections from rats sacrificed at 4 days after gene transfer with each of these vector stocks contained numerous X-gal positive cells proximal to the injection sites. High power views revealed that some of these cells contained neuronal morphology, including positive cell bodies with proximal processes, and more distal positive processes that did not appear to be associated with a cell body were also observed (pNFHlac/ul13g, Figure 1A; pNFHlac/VP16*in*14, gene12, Figure 1D; pNFHlac/Δul46&47, ul13g, Figure 1G; pNFHlac/VP16*in*14, gene12, ul13g, Figure 1J). HSV-1 is known to infect axon terminals and be retrogradely transported to the cell body [23], and small numbers of positive cells were observed at distant sites, including specific areas of neocortex, that contain neurons that project to the striatum (not shown). Subsequent analyses focused on the X-gal positive striatal cells, the majority of the X-gal positive cells.

To determine the efficiency of gene transfer, we quantified the number of positive cells at 4 days after gene transfer divided by the amount of vector that was injected. To quantify any changes in the efficiency of gene transfer caused by packaging using specific mutated HSV-1 protein(s), we compared the efficiencies of gene transfer supported by pNFHlac packaged using specific mutated HSV-1 protein(s) to the efficiency of gene transfer supported by pNFHlac/wt (relative efficiency of gene transfer). Additionally, this ratio should correct for any underestimation of the titers of these vector stocks due to the use of fibroblast cells in the tittering (see methods). The results (Table 1) showed that pNFHlac/Δul46&47, ul13g supported a 5.7-fold increase in the relative efficiency of gene transfer, and we previously observed that pNFHlac/Δul46&47 supported a 9.9-fold increase [19]. Vector stocks that were packaged using either VP16*in*14, gene12 or ul13g, or both, supported modest, if any, increases in the efficiency of gene transfer, similar to previous results from packagings that used either VP16*in*14 or ul13g [14]. In comparing two specific packaging conditions, the efficiency of gene transfer in the striatum may not correlate with the titers obtained using cultured fibroblast cells because of specific aspects of striatal anatomy and physiology, such as the extracellular matrix, that are absent from the cultures of fibroblast cells.

**Figure 2:**
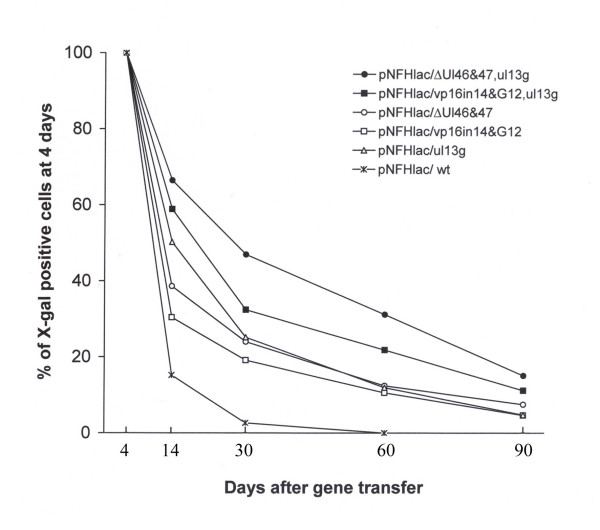
**The stabilities of expression in the striatum supported by pNFHlac packaged using specific combinations of mutated HSV-1 proteins**. For each time point and packaging condition, the graph shows the % of X-gal positive cells at 4 days (mean number of X-gal positive cells at 2 weeks, or 1, 2, or 3 months after gene transfer divided by the mean number of positive cells at 4 days (× 100); calculated using the data in Table 1).

To quantify long-term expression, we compared the numbers of X-gal positive cells at 2 weeks, or 1, 2, or 3 months after gene transfer to the numbers of positive cells at 4 days. This comparison should be independent of any variability in either the titering procedure or the gene transfer process, because this calculation uses the number of positive cells at 4 days for the initial value in the comparison. For the control condition, pNFHlac/wt, by 2 weeks after gene transfer the numbers of X-gal positive cells had declined to 14% of the numbers of positive cells observed at 4 days, by 1 month the number of positive cells was <3% of that at 4 days, and at 2 or 3 months no positive cells were observed (Table 1, Figure 2). {The data using pNFHlac/wt and pNFHlac/Δul46&47, from 4 days through 2 months, was collected in parallel with another study and has been previously reported [19].} The decline in expression we observed with pNFHlac/wt is similar to that we have reported in two previous studies that examined pNFHlac/wt [1,7].

Vector stocks that were packaged using each of the three mutated HSV-1 proteins alone supported X-gal positive cells at 2 or 3 months after gene transfer (pNFHlac/ul13g, Figure 1B and 1C; pNFHlac/VP16*in*14, gene12, Figure 1E and 1F), times when no positive cells were observed using pNFHlac/wt. Cell counts showed that packaging using each of the three mutated HSV-1 proteins alone supported increases in % of X-gal positive cells at 2 weeks, or 1, 2, or 3 months after gene transfer compared to pNFHlac/wt (Table 1, Figure 2). At 2 weeks after gene transfer, the stabilities of expression supported by these 3 packaging conditions ranged from ~30 to 50%, but these 3 packaging conditions supported similar stabilities of expression at 1, 2, or 3 months. The stabilities of expression observed using pNFHlac/ul13g or pNFHlac/VP16*in*14, gene12 were similar to those observed in a previous study that used pNFHlac/ul13g or pNFHlac/VP16*in*14 [14].

The two stocks of pNFHlac that were prepared using specific combinations of mutated HSV-1 proteins supported X-gal positive cells at 2 or 3 months after gene transfer (pNFHlac/Δul46&47, ul13g, Figure 1H and 1I; pNFHlac/VP16*in*14, gene12, ul13g, Figure 1K and 1L). Interestingly, the two vector stocks that were packaged using specific combinations of mutated HSV-1 proteins supported higher percentages of long-term expression than those supported by pNFHlac packaged using each of the mutated HSV-1 proteins alone (Table 1, Figure 2). pNFHlac/Δul46&47, ul13g supported somewhat higher percentages of long-term expression than pNFHlac/VP16*in*14, gene12, ul13g at each of the time points examined. Nonetheless, the percentages of long-term expression exhibited declines during the period that was examined (2 weeks to 3 months) for each packaging condition (Table 1, Figure 2).

We determined the statistical significance of the changes in the percentages of long-term expression using a two way analysis of variance (ANOVA). The ANOVA showed a statistically significant effect of packaging condition (p < 0.001). Subsequent pairwise comparisons showed that the pNFHlac/Δul46&47, ul13g condition supported significantly higher levels of long-term expression compared to each of the other five conditions, including pNFHlac/VP16*in*14, gene12, ul13g, at each of the four time points (12 of these 20 comparisons p < 0.001; 6 of the comparisons p < 0.05; 1 comparison p = 0.056; 1 comparison p > 0.10). Also, subsequent pairwise comparisons suggested that the pNFHlac/VP16*in*14, gene12, ul13g condition supported higher levels of long-term expression compared to each of the three conditions that used a single mutated HSV-1 protein, at each of the four time points (7 of these 12 comparisons p < 0.05; 4 of the comparisons p < 0.10; 1 comparison p > 0.10). Additionally, the ANOVA showed a statistically significant effect of time (p < 0.001). Subsequent pairwise comparisons showed that the percentages of long-term expression declined for comparing each time point to the previous time point, for each packaging condition (p < 0.05).

### The levels of neuronal-specific expression supported by these vector stocks

**Figure 3:**
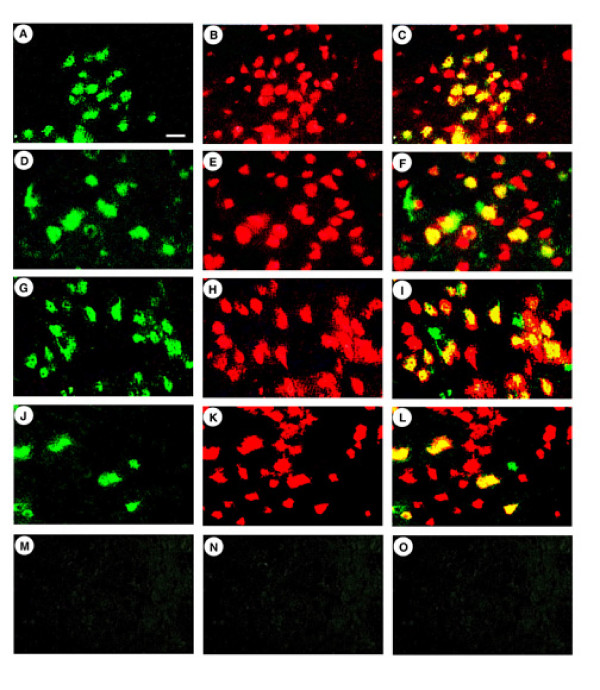
**β-gal-IR positive striatal cells that also contain NeuN-IR from rats sacrificed at 4 days or 2 months after gene transfer with pNFHlac packaged using specific combinations of mutated HSV-1 proteins**. β-gal-IR was detected using a rabbit anti-E. coli β-gal antibody, and was visualized using a fluorescein isothiocyanate-conjugated goat anti-rabbit IgG. In the same sections, NeuN-IR was detected using a mouse monoclonal anti-NeuN, and was visualized using a rhodamine isothiocyanate-conjugated goat anti-mouse IgG. NeuN is a neuronal marker found in the nucleus. **A-C**. pNFHlac/Δul46&47, ul13g, rat sacrificed at 4 days; β-gal-IR (A), NeuN-IR (B), merged (C). Many of the β-gal-IR cells also contain NeuN-IR. D-F. pNFHlac/Δul46&47, ul13g, rat sacrificed at 2 months; β-gal-IR (D), NeuN-IR (E), merged (F). **G-I**. pNFHlac/VP16in14, gene12, ul13g, rat sacrificed at 4 days; β-gal-IR (G), NeuN-IR (H), merged (I). **J-L**. pNFHlac/VP16in14, gene12, ul13g, rat sacrificed at 2 months; β-gal-IR (J), NeuN-IR (K), merged (L). **M-O**. pNFHlac/VP16in14, gene12, ul13g, rat sacrificed at 4 days; no primary antibodies. Scale bar: 25 μm.

Because a specific altered vector particle might potentially support a different cell type specificity of transduction and/or expression than wt vector particles, we confirmed that each of these vector stocks targeted expression to neurons. We performing double staining using antibodies against either *E. coli *β-galactosidase (β-gal) or a neuronal marker, NeuN, on rats sacrificed at 4 days after gene transfer (Table 2; pNFHlac/Δul46&47, ul13g, Figure 3A–C; pNFHlac/VP16*in*14, gene12, ul13g, Figure 3G–I). Additionally, to establish long-term, neuronal-specific expression, we performed the same assay on rats that received either pNFHlac/Δul46&47, ul13g or pNFHlac/VP16*in*14, gene12, ul13g and were sacrificed at 2 months after gene transfer (Figure 3D–F and 3J–L, Table 2). Cell counts showed that each of these vector stocks supported ~90% neuronal specific expression at 4 days after gene transfer, and pNFHlac packaged using each combination of mutated HSV-1 proteins supported ~90% neuronal specific expression at 2 months after gene transfer (Table 2).

**Table 2:** The numbers of β-gal-IR positive cells that costain for NeuN-IR from rats sacrificed at 4 days or 2 months after microinjection of pNFHlac packaged using specific combinations of mutated HSV-1 proteins

Packaging condition	Time after gene transfer	Total β-gal-IR positive cells	β-gal-IR and NeuN-IR positive cells	% costained cells
Δul46&47, ul13g	4 days	199	180	90
Δul46&47, ul13g	2 months	110	98	89
VP16in14, gene12, ul13g	4 days	200	181	91
VP16in14, gene12, ul13g	2 months	78	69	88
VP16in14, gene12	4 days	205	190	93
Δul46&47	4 days	195	179	92
ul13g	4 days	211	189	90
wt	4 days	198	176	89

β-gal-IR was detected using a rabbit anti-*E. coli *β-gal antibody that was visualized with a fluorescein isothiocyanate-conjugated goat anti-rabbit IgG, and NeuN-IR was detected in the same sections using a mouse monoclonal anti-NeuN antibody that was visualized with a rhodamine isothiocyanate-conjugated goat anti-mouse IgG.

## Discussion

HSV-1 gene expression is hypothesized to shut off recombinant gene expression from HSV-1 vectors. However, using this helper virus-free HSV-1 vector system, expression from many promoters remains short-term, even though this vector system does not contain any HSV-1 genes. To resolve this paradox, we hypothesized that specific HSV-1 proteins in the vector particle shut off recombinant gene expression; in support of this hypothesis, we previously reported that packaging using specific mutated HSV-1 proteins improves long-term expression [14]. These results suggested the hypothesis that specific proteins in the HSV-1 particle act by multiple pathways to reduce recombinant gene expression; thus, interfering with multiple pathways might support higher levels of recombinant gene expression than those obtained by interfering with a specific single pathway. The data in this study support this hypothesis and suggest that multiple HSV-1 proteins act together to shut off recombinant gene expression by at least two different pathways. One pathway contains the U_L_13 protein kinase, and a second pathway contains the VP16 transcriptional complex. Of note, the preferred packaging condition was pNFHlac packaged using mutated U_L_13, U_L_46, and U_L_47.

### Titers

Vector particles produced by packaging using a specific mutated HSV-1 protein(s) might have a reduced capability to bind to and/or enter cells. A reduction in the transduction efficiency would likely be detected as an increased ratio of vector genomes (VG) to biological titer (infectious vector particles (IVP)), for a specific packaging condition. In contrast, the VG/IVP ratio was similar for the standard condition (pNFHlac/wt) and each packaging condition that used a mutated HSV-1 protein, including the two conditions that used specific combinations of mutated HSV-1 proteins. Thus, the vector particles produced by these different packaging conditions appear to have similar transduction efficiencies. Similarly, we previously found that packaging vectors in the presence of mutations in each of seven different HSV-1 proteins (U_L_13, vhs, U_L_46, U_L_47, VP16, U_S_3, or U_S_11) did not affect the transduction efficiency [14,19].

The two vector stocks that were prepared using specific combinations of mutated HSV-1 proteins contained sufficient titers to support gene transfer into the rat brain. When used individually, the mutated U_L_13 had modest effects on the titers, and the mutated U_L_46 and U_L_47 had little or no effects on the titers, in either vector packaging or the growth of HSV-1 viruses that contain each mutation [14,16,17]. Consistent with these observations, the pNFHlac/Δul46&47, ul13g condition supported titers that were similar to the standard packaging condition (pNFHlac/wt). In contrast, packaging pNFHlac in the presence of a mutated VP16 reduced the titers ~100-fold [14], and a HSV-1 virus that contained this mutated VP16 displayed reduced growth [22]. Use of EHV-1 gene 12 was previously shown to improve the growth of HSV-1 viruses that contain a mutated VP16 [20]. Analogous to those results, pNFHlac packaged using both a mutated VP16 and EHV-1 gene 12, and either wt or mutated U_L_13, yielded titers that were within an order of magnitude of the titers obtained using the standard packaging condition.

### Improved gene expression from pNFHlac packaged using combinations of mutated HSV-1 proteins

We previously found that packaging pNFHac in the presence of either a mutated U_L_13 or specific components of the VP16 transcriptional complex improved long-term expression [14,19]. There is no known interaction between U_L_13 and the VP16 transcriptional complex. Thus, these observations suggested the hypothesis that there are at least two pathways by which specific proteins in the HSV-1 particle reduce recombinant gene expression. Thus, packaging vectors in the presence of both mutated U_L_13 and a mutated component of the VP16 complex would further improve long-term expression, compared to previous results [14,19].

The results of this study support this hypothesis. pNFHlac packaged using either of two combinations of mutated proteins that included both mutated U_L_13 and a mutated component of the VP16 complex supported higher levels of long-term expression than the levels supported by pNFHlac packaged using any of these mutated HSV-1 proteins, individually. The longest time point examined in this study was 3 months, because that time was sufficient to establish improved long-term expression; however, we have previously shown that HSV-1 vectors support long-term expression for at least 14 months [2]. Interestingly, pNFHlac/Δul46&47, ul13g supported a higher level of long-term expression than pNFHlac/VP16*in*14, gene12, ul13g. This observation is difficult to explain in the absence of more detailed mechanistic information about how U_L_46, U_L_47, and VP16 interact. pNFHlac/Δul46&47, ul13g also improved the efficiency of gene transfer, consistent with previous results with pNFHlac/Δul46&47. The capability of more complex packaging conditions to further improve recombinant gene expression, as shown here, is encouraging for future approaches to obtain additional improvements in expression.

### Potential mechanisms that reduce recombinant gene expression from HSV-1 vectors

Currently available information suggests that U_L_13 and VP16 may act by different mechanisms to reduce recombinant gene expression. In regards to U_L_13: Substrates for U_L_13 include a number of HSV-1 proteins (ICP0, ICP22, gE, and VP22 (U_L_49) [24-27]) and at least two cellular proteins, elongation factor 1δ [28] and RNA polymerase II [29,30]. U_L_13 and cdc2 target the same site in elongation factor 1δ [31]. In regards to VP16: The HSV-1 ori_s _fragment in pNFHlac contains the immediate early (IE) 4/5 promoter and adjacent TAATGARAT elements [7], binding sites for the VP16 transcriptional complex [32]. Although pNFHlac contains three polyadenylation sites between the IE and NFH promoters, increased transcription from the IE 4/5 promoter might interfere with transcription from the NF-H promoter. Alternatively, wt HSV-1, but not *in*1814, activates the c-jun N-terminal kinase/stress-activated protein kinase [33], raising the possibility that this signaling pathway might reduce the activity of the NF-H promoter in pNFHlac.

Although packaging using both a mutated U_L_13 and a mutated component of the VP16 transcriptional complex improved recombinant gene expression, the levels of expression still declined over time, suggesting that additional, as yet unknown, factors contribute the decline in expression. The tegument in the HSV-1 virus particle contains ~15 proteins, and 7 of these proteins have been examined for effects on recombinant gene expression. Two tegument proteins had minimal effects on expression, and specific mutations in each of five tegument proteins improved the efficiency of gene transfer and/or long-term expression [14,19]. It remains to be determined if other tegument proteins affect recombinant gene expression. Other potential factors that might reduce recombinant gene expression include specific proteins in the HSV-1 vector particle capsid or envelope, or the transduction–vector particle disassembly (uncoating) process may be detected by specific cellular signaling pathways.

## Conclusion

In conclusion, the preferred combination of mutated HSV-1 proteins was mutated U_L_13, U_L_46, and U_L_47. Vectors packaged using this combination of mutated proteins supported a higher efficiency of gene transfer and high levels expression for 3 months, the longest time examined. These advances will benefit specific gene therapy applications of HSV-1 vectors.

## Methods

### Materials

Restriction endonucleases were obtained from New England Biolabs and Roche. G418, Dulbecco's modified minimal essential medium, fetal bovine serum, OPTI-MEM I, and lipofectamine were obtained from Invitrogen. X-Gal was purchased from Sigma. Rabbit anti-E. coli β-gal antibody was purchased from ICN, and mouse monoclonal anti-NeuN [34] was from Chemicon. Fluorescein isothiocyanate-conjugated goat anti-rabbit immunoglobulin (Ig) G and rhodamine isothiocyanate-conjugated goat anti-mouse IgG were from Jackson ImmunoResearch Laboratories.

### Vectors, plasmids, and cosmids

pNFHlac [7] contains the mouse NF-H promoter (0.6 kb fragment [35]). pNFHlac supports only short-term expression following packaging using standard conditions (no mutated HSV-1 proteins); rats sacrificed at 4 days after gene transfer contained many X-gal positive cells, but few, if any, positive cells were observed in rats sacrificed at 1 or 2 months [1,7]. Plasmid pC12 contains EHV-1 gene 12 under the control of the cytomegalovirus IE promoter ([20] gift from Dr. Coffin).

Cosmid set C (cos6, cos14, cos28, cos48, cos56, [36]) represents the HSV-1 genome, and the **a **sequence was deleted from the two cosmids that contain it (cos6Δ**a**, cos48Δ**a **[12]). cos6Δ**a**/ul13g [14] contains a point mutation in U_L_13 that abolishes protein kinase activity [14]; HSV-1 particles contain U_L_13 [27,37]. cos56/vp16in14 [14] contains a linker insertion mutation in VP16 [21,22] that abolishes enhancer activity but supports assembly of HSV-1 particles. cos56/Δul46/47 [19] contains a deletion of both U_L_46 and U_L_47 [17]; U_L_46 and U_L_47 are components of VP16 transcriptional complex and are present in HSV-1 particles [16,17,32].

### Cells, vector packaging, and titering

The culture conditions for BHK21 cells or 2-2 cells [38] have been described [14]. Helper virus-free packaging [12] was performed using a modified protocol [39] that improves the titers and cosmids that contain specific mutated HSV-1 proteins, or no mutated HSV-1 proteins (standard or wt conditions). Packaging procedures that used EHV-1 gene 12 employed the standard DNA transfection conditions [12,39] with a 1:1 (μg:μg) ratio of pNFHlac and pC12 [20]. Packaging using VP16in14 reduces the titers ~100-fold [14], and EHV-1 gene 12 was added to improve the titers. Vector stocks were purified as described [40].

The titers of vector genomes (VG)/ml were determined by extracting DNA from the vector stocks and determining the amounts of pNFHlac DNA using PCR and primers that recognize the Lac Z gene [14]. The titers of infectious vector particles (IVP)/ml were determined by quantifying the numbers of X-gal positive cells at 1 day after transduction of BHK cells [14]. Expression from the NF-H promoter (in pNFHlac) in BHK fibroblast cells is ectopic expression, and this ectopic expression declines at longer times after gene transfer [1,14]. We used this fibroblast cell line rather than a neuronal cell line, such as PC12 cells, because we previously found that titering pNFHlac stocks on PC12 cells results in lower titers compared to those obtained on BHK fibroblast cells [1,14], possible because PC12 cells do not form a monolayer that facilitates efficient transduction and accurate titering. wt HSV-1 was not observed in these vector stocks (<10 plaque forming units/ml).

### Expression experiments in the rat brain

These studies were approved by the W. Roxbury VA Hospital IACUC. Stereotactic injections (3 μl/site, 2 sites) were used to deliver vector stocks into the striatum of male Sprague Dawley rats (150–175 gm; anterior-posterior (AP) +0.8, medial-lateral (ML) +2.5, dorsal-ventral (DV) -5.5; AP +0.8, ML -2.5, DV -5.5). AP is relative to bregma, ML is relative to the sagittal suture, and DV is relative to the bregma-lambda plane [41]. Vector stocks were injected using a micropump (model 100, KD Scientific); 3 μl of vector stock was injected over 8 minutes, the needle was maintained in place for an additional 5 minutes, and the needle was slowly withdrawn over approximately 5 minutes. At 4 days to 3 months after gene transfer, the rats were perfused, the brains were sectioned, and X-gal staining was performed [1] at room temperature, pH 7.9, for 3 hours. Alternatively, to localize β-gal to neurons, rabbit anti-*E. coli *β-gal was visualized with a fluorescein isothiocyanate-conjugated goat anti-rabbit IgG, and mouse monoclonal anti-NeuN was visualized with a rhodamine isothiocyanate-conjugated goat anti-mouse IgG [1].

### Cell counts

Coronal sections (25 μm) that included the striatum were prepared. Every 4th section was analyzed for recombinant gene expression, and ~12 of these sections contained either the X-gal or the β-gal-immunoreactivity (IR) positive cells. Low power (10×) was used to identify the positive cells, and high power (40×) was used to perform the cell counts. To verify the accuracy of the cell counts, each section was counted at least two times, on different days, and the values differed by <10% for each section. ANOVA followed by multiple pairwise comparisons was used to analyze the statistical significance of differences in the numbers of X-gal cells.

## Abbreviations

ANOVA: analysis of variance; AP: anterior-posterior; β-gal: β-galactosidase; BHK: baby hamster kidney; DV: dorsal-ventral; EHV-1: equine herpesvirus; HSV-1: Herpes Simplex Virus; IE: immediate early; Ig: immunoglobulin; IR: immunoreactivity; IVP: infectious vector particles; LAT: latency associated transcript; ML: medial-lateral; NF-H: neurofilament heavy gene; TH: tyrosine hydroxylase; VG: vector genomes; wt: wild type; X-Gal: 5-bromo-4-chloro-3-indoyl-β-D-galactopyranoside.

## Authors' contributions

ML performed the vast majority of this study, and was assisted in the vector packaging by XL. AG wrote the manuscript. AG conceived of the study, and participated in its design and coordination. All authors read and approved the final manuscript.
